# T cells and reactive oxygen species

**DOI:** 10.1186/s12929-015-0194-3

**Published:** 2015-10-15

**Authors:** Aleksey V. Belikov, Burkhart Schraven, Luca Simeoni

**Affiliations:** Otto-von-Guericke University, Universitätsplatz 2, 39106 Magdeburg, Germany; Institute of Molecular and Clinical Immunology, Otto-von-Guericke University, Leipziger Str. 44, Magdeburg, 39120 Germany

**Keywords:** T lymphocytes, ROS, Oxidative stress, Hyporesponsiveness, AICD, Apoptosis, Activation, Differentiation, Signaling, TCR

## Abstract

*Reactive oxygen species* (ROS) have been long considered simply as harmful by-products of metabolism, which damage cellular proteins, lipids, and nucleic acids. ROS are also known as a weapon of phagocytes, employed against pathogens invading the host. However, during the last decade, an understanding has emerged that ROS also have important roles as signaling messengers in a multitude of pathways, in all cells, tissues, and organs. T lymphocytes are the key players of the adaptive immune response, which both coordinate other immune cells and destroy malignant and virus-infected cells. ROS have been extensively implicated in T-cell hyporesponsiveness, apoptosis, and activation. It has also become evident that the source, the kinetics, and the localization of ROS production all influence cell responses. Thus, the characterization of the precise mechanisms by which ROS are involved in the regulation of T-cell functions is important for our understanding of the immune response and for the development of new therapeutic treatments against immune-mediated diseases. This review summarizes the 30-year-long history of research on ROS in T lymphocytes, with the emphasis on the physiological roles of ROS.

## Introduction to ROS

ROS are small short-lived oxygen-containing molecules that are chemically highly reactive, a property that is mainly due to their unpaired electrons (radicals). *Superoxide* (O_2_^•−^), *hydrogen peroxide* (H_2_O_2_), hydroxyl radical (OH^•^), hypochlorous acid (HOCl), lipid peroxides (ROOH), singlet oxygen (^1^O_2_), and ozone (O_3_) are some of the most common ROS [1]. The first two species are the most important ROS involved in the regulation of biological processes. O_2_^•−^ is usually the species from which other ROS originate. Once produced, O_2_^•−^ either rapidly reacts with surrounding molecules or dismutates to H_2_O_2_, spontaneously or with the help of *superoxide dismutase* (SOD) [2]. H_2_O_2_ is more stable, less reactive, can diffuse in the microenvironment and even cross cell membranes. H_2_O_2_ can either react with particular amino acids, usually cysteines and methionines, or can be converted to OH^•^ (in the Fenton reaction), HOCl (by myeloperoxidase), or H_2_O (with the help of *catalase*, *peroxidase*, or *peroxiredoxin*) [3]. Both OH^•^ and HOCl are highly reactive and usually irreversibly damage nearby molecules.

One of the major sources of ROS in the cell are mitochondria [4, 5]. Mitochondria express the *electron transport chain* (ETC.) complexes, which transfer electrons from NADH and succinate, along a controlled redox pathway, to the oxygen molecule (O_2_). Upon receiving four electrons, O_2_ is reduced to H_2_O. However, the ETC is not perfect, and occasionally O_2_ undergoes one- or two-electron reduction to form O_2_^•−^ or H_2_O_2_, respectively. *Complexes I* and *III* of the ETC are the main sources of mitochondrial O_2_^•−^ [4, 5]. Multiple metabolic enzymes, such as ERO-1, cytochromes P-450 and b5, lipoxygenases, cyclooxygenases, α-ketoglutarate- and glycerol phosphate dehydrogenases, as well as hydroxyacid-, urate-, xanthine-, monoamine-, diamine-, polyamine-, and amino acid oxidases, are also producing ROS as necessary intermediates or byproducts of their reactions [6]. These enzymes can be found in mitochondria, endoplasmic reticulum, peroxisomes, and cytosol. There is also a large class of ROS producing enzymes called *NADPH oxidases* (see below). Finally, there are exogenous sources of ROS, including ultraviolet and gamma radiation, smoke and other air pollutants, as well as several drugs and chemicals. As ROS can damage proteins, lipids, and nucleic acids, the evolution has created specialized antioxidant systems. There are antioxidant enzymes, such as SODs, catalases, *glutathione peroxidases* (GPXs), *peroxiredoxins* (PRXs), *thioredoxins* (TRXs), glutaredoxins (GRXs), sulfiredoxins (SRXs), thioredoxin reductases, glutathione reductases, and methionine sulfoxide reductases [7], and also small nonenzymatic antioxidant molecules, such as *glutathione*, *ascorbate*, pyruvate, α-ketoglutarate, and oxaloacetate [8]. When the rate of ROS production in the cell (or in the microenvironment) significantly exceeds the rate of their neutralization by the antioxidant systems, the cell undergoes *oxidative stress*. Prolonged or excessive oxidative stress can lead to the impairment of cellular functions, cell death, senescence, or malignant transformation [9–11].

Phagocytic cells produce ROS to kill engulfed bacteria during the so-called *respiratory burst* [12]. In these cells, ROS are produced by the *phagocytic NADPH oxidase* (PHOX), an enzyme consisting of several subunits [13]. The catalytic subunit, called the *PHOX glycoprotein of 91 kDa* (gp91^phox^) or the *NADPH oxidase 2* (NOX-2), is expressed at either the plasma or phagosomal membrane. PHOX includes also a variety of regulatory subunits: membrane-anchored p22^phox^ and cytoplasmic p40^phox^, p47^phox^, and p67^phox^, as well as the RAC GTPase. PHOX becomes functional upon the tightly regulated assembly of this multisubunit complex. Interestingly, six homologs of gp91^phox^ (NOX-2) have been identified in different tissues: NOX-1, NOX-3, NOX-4, NOX-5, *dual oxidase 1* (DUOX-1), and DUOX-2 [14, 15]. NOXs are usually activated upon the triggering of cell receptors by their respective ligands, such as insulin, angiotensin, PDGF, GM-CSF, TNF, chemokines that bind G protein-coupled receptors, complement component 5a (C5a), lysophospholipids, and leukotriene B4, as well as by cell adhesion and by phagocytosis [8]. Because of the widespread yet differential expression of NOX and DUOX isoforms across organelles, cell types, and organisms, O_2_^•−^ and H_2_O_2_ can be considered as ubiquitous signaling messengers. Indeed, during the last decade, it has become evident that ROS are not just harmful byproducts of metabolism and weapons of phagocytes but are also crucial players in cellular signaling. ROS-mediated signaling is involved in multiple processes, such as cell growth [14, 15], stem cell renewal [16, 17], tumorigenesis [8, 14, 17], cell death [14, 15], cell senescence [15, 17], cell migration [16], oxygen sensing [15], angiogenesis [15], circadian rhythm maintenance [16], and immune responses [8, 15].

Among ROS, H_2_O_2_ acts as the major signaling messenger and is excellently suited for this function [18]. In fact, it is stable enough, is able to cross cell membranes, and is reacting preferentially with cysteine residues [3]. It has to be noted that cysteine residues are amongst the most conserved and least abundant protein residues [19], which ensures high selectivity and specificity for oxidation-mediated post-translational modifications. Moreover, only specific cysteinyl thiols that, upon coordination with neighboring amino acid side chains, can become thiolate anions are able to react with H_2_O_2_ [20]. When H_2_O_2_ oxidizes a cysteine thiolate anion (R-S^−^), sulfenic acid (R-SOH) is formed [21, 22]. This process, which is referred to as *sulfenylation*, is reversed by GRXs and TRXs [7, 20]. Thus, sulfenylation is believed to be akin to phosphorylation or other post-translational modifications. Sulfenylation may lead to further post-translational modifications, such as glutathionylation, disulfide bond formation, and sulfinilation [21, 22]. Most importantly, it can be involved in the regulation of protein activity. In fact, sulfenylation can induce changes in the protein conformation, thus leading to the activation or inactivation of the catalytic center or to other functional alterations of the protein. Multiple protein classes have been shown to be regulated by sulfenylation, including phosphatases and kinases, transcription factors and histone deacetylases, antioxidant enzymes and heat-shock proteins, proteases and hydrolases, ion channels and pumps, adaptor molecules and cytoskeleton components [8, 16, 17, 21–23].

It is believed that the NOX enzymes are the major source of *signaling ROS* [24]. Nevertheless, it is likely that ROS produced by the mitochondrial ETC or metabolic enzymes are also involved in signaling processes [25–28]. The levels of ROS that are involved in signal transduction are much lower than the levels occurring during respiratory burst or oxidative stress. Moreover, due to the abundance of antioxidant systems in the cell, ROS cannot travel long distances, and hence they transmit signals only locally, in confined compartments [29, 30]. In other words, the source and the corresponding targets of signaling ROS usually have to be in close proximity. ROS-mediated signaling can be additionally regulated via controlled alterations in local levels and activity of specific antioxidants [16, 23]. For example, glutathione is a good scavenger for many ROS, such as HOCl, but reacts too slowly with H_2_O_2_, the major mediator of oxidative signaling. On the contrary, PRXs have remarkably high reaction rates with H_2_O_2_, and their activity is tightly regulated by phosphorylation and sulfinilation. In fact, PRXs appear to be the major scavengers of signaling ROS, akin to phosphatases in the kinase-phosphatase system, and thus constitute a crucial component of redox signaling [7, 16, 23, 31–34]. Moreover, some members of aquaglyceroporin and aquaporin protein families can enhance the permeability of cell membranes to H_2_O_2_, thus providing an additional level of regulation to ROS-mediated signaling [16].

Thus, the complex role of ROS in T-cell biology can be simplified by dividing it in two parts: (i) the effects of large exogenous quantities of ROS, known as oxidative stress, and (ii) the function of compartmentalized, dose- and time-controlled endogenous ROS (hereafter referred to as signaling ROS). The latter can be implicated in both the activation and the apoptosis of T cells. This review covers all three aspects of ROS involvement in T-cell functions.

## Review

### T cells and oxidative stress

**Fig. 1:**
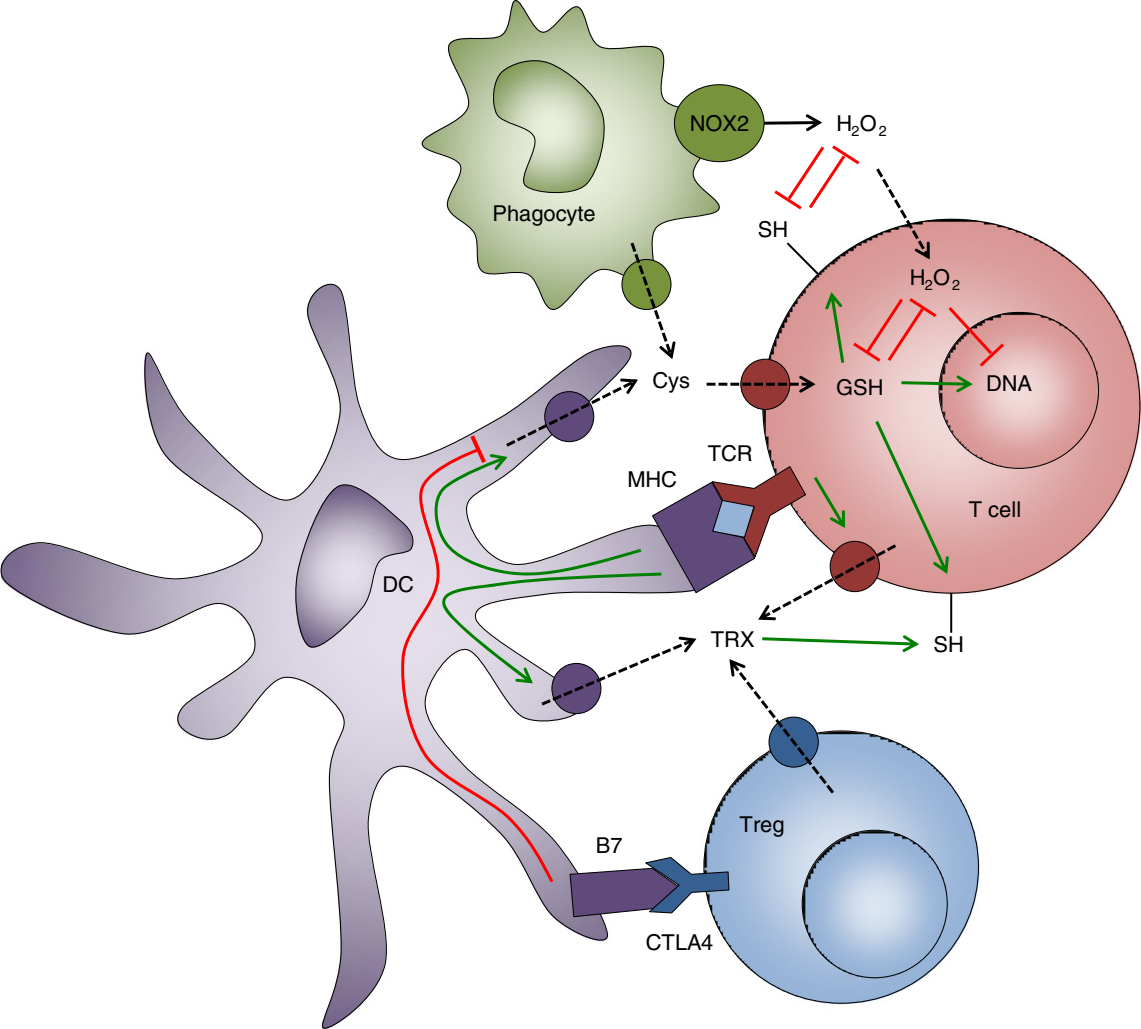
The redox regulation of T-cell state. Activated phagocytes produce H_2_O_2_ via NOX-2. H_2_O_2_ either oxidizes thiols (SH-) on the surface of T cells or enters inside T cells. Intracellularly, H_2_O_2_ either oxidizes glutathione (GSH) or interferes with DNA synthesis. Activated phagocytes and dendritic cells (DC) secrete cysteine (Cys) to the extracellular space. Cys is taken up by T cells and converted to GSH. GSH keeps surface thiols in the reduced state, neutralizes intracellular H_2_O_2_, and enables DNA synthesis. TCR-peptide-MHC interaction leads to the secretion of thioredoxin (TRX) by T cells, DCs, and T_regs_. TRX helps to keep surface thiols in the reduced state. Black solid arrow indicates production, black dashed arrows indicate import/export, green solid arrows indicate activation, red bar-headed lines indicate inhibition.

T cells are often present in close proximity to phagocytic cells, which are known to produce large amounts of ROS during respiratory burst. Moreover, activated T cells can trigger respiratory burst by direct contacts with phagocytes [35], as well as by secreted cytokines. The phagocyte-produced ROS can reach T cells and cause oxidative stress (Fig. 1). For example, it has been shown that activated neutrophils inhibit DNA synthesis in human T cells proportionally to superoxide levels in the medium (measured by cytochrome c reduction) [36]. More importantly, the treatment with the glutathione precursor *N-acetyl cysteine* (NAC) or catalase prevents the inhibition [36]. Further analysis showed that this impairment of DNA synthesis is associated with alterations in the T-cell receptor (TCR) signaling, including conformational changes in TCRζ and LCK, reduced PLCγ-1 phosphorylation and calcium flux, and increased ERK phosphorylation [36]. Nevertheless, this study showed that activated neutrophils do not induce apoptosis in T cells. Interestingly, a different study demonstrated the decreased viability of human CD4^+^ T cells upon coculture with autologous granulocytes, which was reversed by the addition of catalase [37]. Another study attempted to mimic the effects of phagocyte-derived ROS by treating human T cells with polyamine oxidase-generated H_2_O_2_ for prolonged time [38]. This treatment suppressed the tyrosine phosphorylation, calcium flux, NFAT and NFkB (but not AP-1) activation, and IL-2 production [38]. Thus, it can be concluded from these studies that phagocyte- or, at least, neutrophil-derived ROS negatively affect T-cell signaling, activation, proliferation, and, potentially, viability. Whether this mechanism has evolved in order to prevent excessive inflammation at sites of infection is not currently known. Interestingly, to our knowledge, the direct suppression of T-cell responses by macrophage-derived ROS has not been shown yet.

To assess the impact of oxidative stress on T-cell viability, applications of micromolar doses of H_2_O_2_ have been performed. It appears that the susceptibility of human T cells to H_2_O_2_-induced apoptosis strongly depends on the T-cell subset. In fact, 5 μM H_2_O_2_ induces apoptosis in CD45RO^+^ T_mem_ but not in CD45RA^+^ T_nai_ cells, via the mitochondrial depolarization and caspase activation [39]. Similarly, CD4^+^ CD45RA^+^ T_nai_ cells are less likely to undergo cell death upon incubation with 5–20 μM H_2_O_2_ than CD4^+^ CD45RA^−^ T_mem_ cells [37]. In contrast to conventional T cells, T_reg_ cells have lower intracellular ROS levels and are protected from H_2_O_2_-induced death [37]. Moreover, 10 μM H_2_O_2_ does not affect T_reg_ suppressive capacity [37]. Interestingly, 100 μM H_2_O_2_ completely eliminates CD4^+^ T cells but has no effect on CD4^+^ T-cell blasts [40]. Thus, T-cell resistance to exogenous H_2_O_2_decreases in the following order: T_eff_ > T_reg_ > T_nai_ > T_mem_, which may reflect the probability of a given subset to appear near ROS-producing phagocytes in an inflamed environment. It is likely that T_eff_ cells are largely protected from ROS-mediated death and can assist phagocytes in the elimination of pathogens. However, it is unclear whether the application of a single H_2_O_2_ dose, as compared to the continuous production of ROS by phagocytes, has a physiologically meaningful effect on T cells.

Phagocytic cells regulate the redox state of the microenvironment not only by releasing ROS but also by producing antioxidants, especially the glutathione precursor *cysteine* (Fig. 1). In fact, activated murine macrophages secrete cysteine that can be taken up by T cells, resulting in the increased intracellular glutathione level [41]. Dendritic cells (DCs) also can secrete cysteine (Fig. 1). The LPS stimulation or the coincubation of murine DCs with T cells leads to the release of cysteine by DCs, its subsequent uptake by T cells, and conversion to glutathione [42]. This correlates with the acquisition of the reduced state by the T-cell surface thiols [42]. Human DCs also secrete cysteine, as well as thiol-reducing TRX, upon co-incubation with alloreactive T cells [43]. The coculture of antigen-pulsed human DCs with autologous T cells leads to increase in the levels of cell-surface and intracellular thiols in antigen-specific T cells and protects them from H_2_O_2_-induced apoptosis [44]. Thus, activated macrophages and DCs secrete cysteine to the extracellular space, which is taken up by T cells and converted to the antioxidant glutathione. Glutathione helps to counteract the suppressive effects of phagocyte-derived ROS. The interesting question is whether also neutrophils can release cysteine or TRX upon activation, and if not, whether this contributes to the suppressive effects of their respiratory burst on T-cells [36, 37]. It could be possible that neutrophils do not release antioxidants to maximize their antimicrobial killing capacity, during the acute phase of inflammation, whereas macrophages and DCs release cysteine to protect T-cells from ROS during antigen presentation, in late and chronic inflammation.

Interestingly, murine T_regs_ can suppress cysteine release by DCs, leading to the oxidation of surface thiols, decrease in intracellular glutathione, and reduction in DNA synthesis in conventional T cells [42]. The addition of exogenous cysteine partially reverts the T_reg_-induced inhibition of DNA synthesis in murine T cells [45]. Further experiments showed that murine T_regs_ suppress glutathione synthesis and cysteine release by DCs in a CTLA-4-dependent manner [45] (Fig. 1). Thus, T_regs_ can interfere with the cysteine release by DCs and hence allow phagocyte-derived ROS to inhibit T-cell activation. It is crucial to confirm this previously unknown mechanism of suppression in human T_regs_. Interestingly, in contrast to conventional T cells, human T_regs_ have higher thiol content (but express similar levels of catalase, *manganese SOD* (MnSOD, SOD-2) and *copper-zinc SOD* (CuZnSOD, SOD-1)) and are more resistant to cell death induced by granulocyte-secreted H_2_O_2_ [37]. Moreover, human T_regs_ express and secrete more TRX-1 than conventional T cells and upregulate it stronger upon stimulation [46] (Fig. 1). Their ability to suppress T cells by contributing to the formation of an oxidative milieu is thus questionable.

Glutathione is required for DNA synthesis in murine [42, 45, 47] and human [48, 49] T cells, as well as for proper LAT conformation in human T cells [50]. It seems that particular levels of this antioxidant are required to maintain redox balance in T cells, which, in turn, appears to be indispensable for signaling and proliferation. However, it cannot be excluded that T-cell proliferation depends on some redox-unrelated functions of glutathione. Further investigation is required to distinguish between those possibilities.

Overall, under physiological conditions, there is likely an equilibrium between ROS and antioxidant systems, which is required for the proper functioning of T cells (Fig. 1). However, the perturbation of this equilibrium by changing the levels of ROS or antioxidants can result in T-cell hyper- or hyporesponsiveness, which, in turn, may lead to the development of various pathologies. For example, a genetic deficiency in ROS production by macrophages [51] or artificially increased thiol levels in T cells [52] lead to T cell-mediated arthritis upon the collagen immunization of mice. On the contrary, myeloid-derived suppressor cells, found in the majority of cancer patients, inhibit T cells by lowering the amount of cysteine released by macrophages and DCs, at least in mice [53].

### ROS as regulators of activation-induced cell death

**Fig. 2:**
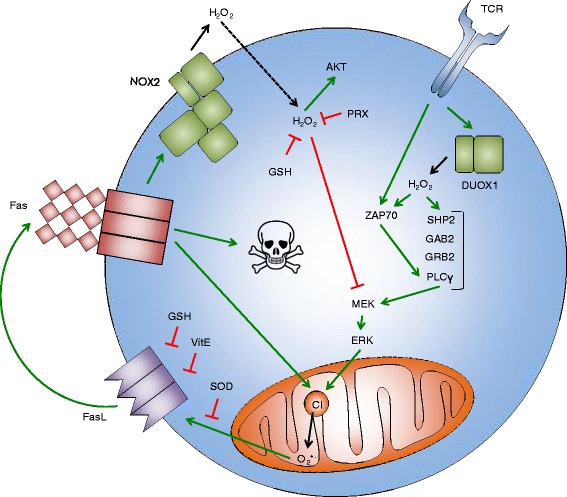
ROS in activation-induced T-cell death. TCR triggering leads to the activation of DUOX-1, which produces H_2_O_2_ that enhances the activation of ZAP-70 and the formation of the SHP-2-GAB-2-GRB-2-PLCγ-1 complex. MEK-ERK pathway increases O_2_^•-^ production by mitochondrial Complex I (CI), which leads to the expression of FasL. Superoxide dismutase (SOD), vitamin E (VitE), and glutathione (GSH) interfere with FasL expression. FasL triggers Fas that initiates apoptosis execution, further enhances O_2_^•-^ production by CI, and activates NOX-2. NOX-2 produces H_2_O_2_ that enters the cell and activates AKT but inhibits MEK. H_2_O_2_ is neutralized by peroxiredoxin (PRX) and GSH. Black solid arrows indicate production, black dashed arrow indicates import, green solid arrows indicate activation, red bar-headed lines indicate inhibition. Skull and bones indicate apoptosis

The stimulation of previously activated T cells – also called *T-cell blasts* – results in *activation-induced cell death* (AICD) [54], a process which is accompanied by the release of ROS. NOX-2, the major ROS-producing enzyme of phagocytes, has been shown to be expressed also in T-cell blasts, albeit at a very low level [55]. The same study showed that the TCR stimulation of murine T-cell blasts induces the Fas-dependent oxidation of the ROS-sensitive dye *2*’,*7*’*-dichlorofluorescein diacetate* (DCFDA) by NOX-2-derived ROS [55] (Fig. 2). Moreover, this stimulation also induces transient DCFDA oxidation that is not dependent on Fas or NOX-2, as well as the Fas-dependent oxidation of the superoxide-sensitive dye *dihydroethidium* (DHE) that is not mediated by NOX-2 [55], indicating that at least two more sources of ROS are involved in AICD. Similarly, in human T-cell blasts, TCR stimulation triggers the MEK-dependent oxidation of DHE and DCFDA [56, 57]. Moreover, TCR-triggered DCFDA oxidation in human CD4^+^ T-cell blasts is dependent on the DUOX-1 [58]. Other group has shown that the TCR stimulation of human T-cell blasts leads to DHE and DCFDA oxidation, FasL expression, and cell death that all depend on mitochondrial Complex I [59–61]. Thus, the ligation of TCR or Fas in T-cell blasts can lead to ROS production via NOX-2, DUOX-1, mitochondria, and possibly other sources (Fig. 2). However, it remains to be shown whether human T-cell blasts produce ROS via NOX-2, and whether murine blasts produce ROS via DUOX-1 or mitochondria, upon TCR triggering.

To understand the function of the different sources of ROS in T-cell blasts, RNA interference and knockout mice have been used. It has been shown that MEK and ERK activation is enhanced, whereas AKT activation is suppressed, in T-cell blasts from NOX-2-deficient mice [55] (Fig. 2). The suppression of DUOX-1 with siRNA in human CD4^+^ T-cell blasts leads to decrease in the ZAP-70, PLCγ-1, and ERK phosphorylation, as well as PLCγ-1, SHP-2, and GAB-2 association with GRB-2 [58] (Fig. 2). Thus, NOX-2 and DUOX-1-derived ROS are involved in TCR-mediated signaling in T-cell blasts, but their effects are different. This might be due to the different localization of NOX-2 and DUOX-1 in T cells and/or the different timing of their activation. Unfortunately, it is not possible to study the function of ROS from mitochondria by performing the knockdown of Complex I, as this will lead to the general impairment of mitochondrial function and will affect cell metabolism.

Various antioxidant compounds and the overexpression of antioxidant enzymes have also been used in order to understand the function of ROS in the signaling and the apoptosis of T-cell blasts (Fig. 2). In agreement with the results from NOX-2-deficient mice [55], both the addition of NAC and the overexpression of PRX-2 sustain MEK and ERK phosphorylation but delay AKT phosphorylation in human T-cell blasts [57]. This indicates that intracellular H_2_O_2_ is the main mediator of the observed NOX-2 effects in T-cell blasts. The overexpression of CuZnSOD or MnSOD (but not catalase or TRX peroxidase) in human T-cell blasts abrogates FasL expression [56], strengthening the evidence for mitochondrial O_2_^•-^ participation in this process [59–61]. Additionally, FasL upregulation and the cell death of human T-cell blasts are prevented by NAC [59, 61] and vitamin E [62].

In summary, it seems that several different sources of ROS are involved in the AICD of T cells (Fig. 2). First, H_2_O_2_ produced by DUOX-1 upon TCR triggering serves to amplify proximal signaling events downstream of the TCR. Next, O_2_^•-^ released from mitochondrial Complex I, potentially in response to ERK signaling, triggers the expression of FasL. Finally, Fas ligation activates NOX-2, which probably assists the execution of the apoptotic program via the H_2_O_2_-mediated activation of AKT and the inhibition of MEK. Moreover, cell-intrinsic antioxidants, such as glutathione, vitamin E, MnSOD, and CuZnSOD, interfere with FasL expression, thus counteracting AICD. A better understanding of the role of ROS in the mechanisms of AICD could help in the development of therapeutic strategies for diseases in which T cells either die excessively or are not properly cleared during the contraction phase of immune response, such as AIDS or autoimmune diseases, respectively. As an example, vitamin E has already been shown to reduce FasL expression and AICD in T cells from HIV-infected patients [62].

### ROS as regulators of the activation of primary T cells

**Fig. 3:**
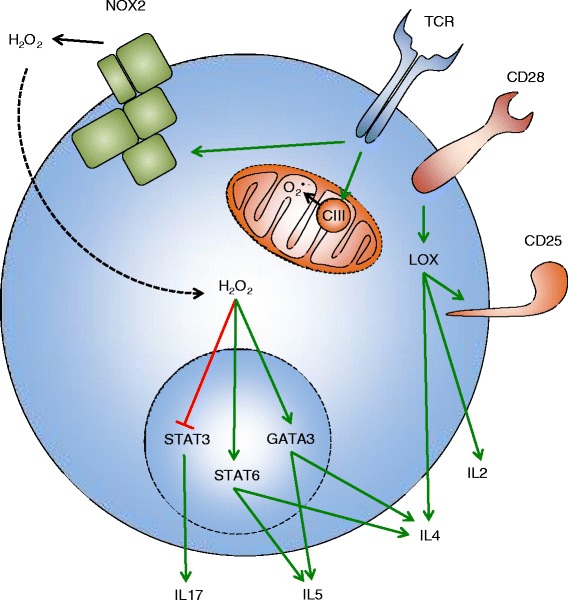
ROS in the activation of primary T cells. The ligation of CD28 leads to the activation of the lipoxygenase pathway (LOX). This enhances the expression of CD25, IL-2, and IL-4. The triggering of TCR leads to increase in O_2_^•-^ production by mitochondrial Complex III (CIII). Whether this O_2_^•-^ has any function is not clear. Additionally, the ligation of TCR leads to the activation of NOX-2, which produces H_2_O_2_ that enters the cell. Inside the cell, H_2_O_2_ activates GATA-3 and STAT-6 but inhibits STAT-3. GATA-3 and STAT-6 direct T-cell differentiation towards the T_h_2 phenotype and the production of IL-4 and IL-5. STAT-3 leads to differentiation into T_h_17 cells and to IL-17 production. Black solid arrows indicate production, black dashed arrow indicates import, green solid arrows indicate activation, red bar-headed line indicates inhibition

There is evidence that primary human and murine T cells produce ROS upon stimulation with different agents. However, it cannot be excluded that the detected ROS originate from contaminating phagocytic cells, which are almost inevitably present in any preparation of primary T cells. Phagocytes can be activated by various agents used to stimulate T cells, for example by PMA or by anti-TCR antibodies via Fc receptors [63]. Desirably, the purity of the T-cell preparation should be very high (>99 %), and an isotype-matched control antibody should be used to discriminate between the T cell-derived ROS and the ROS of phagocytic origin. Unfortunately, these conditions have been rarely met. Moreover, activated T cells can induce respiratory burst in phagocytes by direct contacts [35], so the use of an isotype control is not the ultimate solution to this problem. Thus, the detection of specific T cell-derived ROS is a challenging task. The following paragraph summarizes data showing ROS production in primary human and murine T cells (Fig. 3).

TCR or CD28 stimulation of human T cells induces 5-lipoxygenase-dependent DCFDA oxidation and decreases intracellular glutathione levels [64]. TCRxCD28 stimulation of human or murine T cells induces extracellular O_2_^•-^ production, measured using the luminol-based *Diogenes* system. O_2_^•-^ production is completely abrogated in T-cell preparations from NOX-2-deficient mice [65]. Similarly, TCR stimulation of murine CD4^+^ T cells induces NOX-2-dependent DCFDA oxidation [66]. Thus, these data indicate that NOX-2 is an important source of ROS in T cells. Additionally, TCR or TCRxCD28 (but not CD28) stimulation of murine CD4^+^ T cells induces the oxidation of the *mitochondrial reduction-oxidation sensitive green fluorescent protein* (mito-roGFP). Interestingly, it has been shown that the oxidation of mito-roGFP is dependent on mitochondrial calcium uptake [67]. The same study showed that TCRxCD28 stimulation induces the oxidation of the mitochondria-targeted superoxide-sensitive dye *MitoSOX*. This oxidation is dependent on the mitochondrial Rieske iron-sulfur protein (RISP), a subunit of Complex III [67]. CD8^+^ murine T cells also induce DCFDA oxidation upon TCR or CD28 stimulation [68]. Collectively, the data reported above suggest that the triggering of the TCR and CD28 in primary T cells leads to ROS production by 5-lipoxygenase, NOX-2, and mitochondria. As the majority of the studies have been in performed in murine T cells, their findings have to be confirmed in human cells. However, to ultimately solve the problem of the ROS origin, T cell-specific knockouts of NOX-2 or other ROS-producing enzymes have to be used.

T cells from mice lacking components of NOX-2, such as gp91^phox^ or p47^phox^, could serve as a good model to study the function of NOX-2-derived ROS in T-cell activation. Nevertheless, results obtained using this model should be interpreted with caution, as phagocytes from these mice also lack functional NOX-2, and this may indirectly affect T cells. A T-cell specific knockout of NOX-2 will be helpful. T cells from p47^phox−/−^ mice have the diminished expression of T-bet, STAT-1, and STAT-4 transcription factors and the lowered production of IL-2, IL-4, IFNγ, TNFα, and GM-CSF [66]. Additionally, these cells have the enhanced phosphorylation of STAT-3 and the increased production of IL-10, IL-17, and TGFβ [66]. Overall, the p47^phox^ deficiency leads to differentiation towards the T_h_17 lineage. Interestingly, another study showed that CD4^+^ T cells from gp91^phox−/−^ mice display the T_h_1 phenotype. These cells produce less IL-4 and IL-5 but more IL-17 and IFNγ than the wild-type counterparts [69]. Moreover, they have reduced GATA-3 expression and diminished STAT-5 and STAT-6 phosphorylation but increased T-bet expression [69]. The stark differences in T-bet and IFNγ expression between the two studies might be explained by the different NOX-2 subunits that have been knocked out, probably indicating that in addition to participating in the assembly of PHOX they may have other functions. However, both studies detected decreased IL-4 and increased IL-17 production in NOX-2-deficient cells, indicating conversion from the T_h_2 to the T_h_17 phenotype (Fig. 3). Interestingly, gp91^phox−/−^ T cells have no defect in CD25 expression or IL-2 production [69]. This was confirmed by a recent study, which showed that T cells from gp91^phox−/−^ mice have the normal expression of CD25 and CD69 and normal proliferation [65]. Thus, NOX-2 is required for proper differentiation but not for the activation of primary murine T cells. The confirmation of these findings in human T cells using shRNA or CRISPR would be helpful to understand the function of NOX-2 in humans. However, this is complicated by the low transfectability of primary T cells [70], and by oxidative stress [71] and general activation [72] that will be induced upon electroporation. The elucidation of the role of lipoxygenase- or RISP-derived ROS by the downregulation of these proteins is even more problematic, as this will additionally disturb cell metabolism.

Another approach that has been used to understand the functional importance of ROS in the activation of primary T cells is incubation with antioxidants. In murine T cells, *butylated hydroxyanisole* (BHA) blocks DNA synthesis and CD25 expression [73], whereas the *mitochondria-targeted vitamin E* (Mitovitamin E) abrogates IL-2 production [67]. In human T cells, *nordihydroguaiaretic acid* (NDGA) inhibits IL-2 synthesis [74], whereas vitamin E suppresses IL-4 production [75]. Interestingly, catalase, superoxide dismutase, and ascorbate do not affect the expression of CD25 and CD69, proliferation, and cytokine production in human T cells [65]. It seems that antioxidants that inhibit lipid peroxidation and/or lipoxygenase activity (such as NDGA, BHA, Vitamin E, or Mitovitamin E), but not antioxidants that scavenge water-soluble ROS (such as catalase, superoxide dismutase, or ascorbate) can interfere with the activation of primary T cells (Fig. 3). The intriguing possibility is that reduction in the synthesis of leukotrienes (proinflammatory molecules downstream of lipoxygenase), rather than reduction in ROS levels *per se* [76], could be the reason for the impairment of T-cell activation by lipid-soluble antioxidants. This hypothesis is in agreement with the findings that NOX-2 deficient T cells have normal CD25 and CD69 expression, IL-2 production, and proliferation [65, 69].

Collectively, it seems that ROS produced by NOX-2 are involved in the differentiation of T cells but not in the initial phase of T-cell activation. The further expansion of knowledge on the role of NOX-2 and other ROS sources in primary T-cells will result in the clinical applications. For example, it has been recently shown that T cells from patients with systemic sclerosis express elevated levels of NOX-2 and have higher basal ROS levels [77].

## Conclusions and perspectives

T cells are not isolated entities and are present in various tissue microenvironments. It has become evident that the surrounding cells create a particular redox milieu that may, in turn, influence T-cell responses. For example, neutrophils in the inflamed tissues produce large amounts of ROS, which can suppress or even kill T cells. On the contrary, macrophages and dendritic cells secrete the glutathione precursor cysteine and the thiol-reducing enzyme thioredoxin, which increase the oxidation resistance of T cells. Moreover, T_regs_ can interfere with these processes and hence regulate T-cell activation. Additionally, appropriate intracellular ROS levels in T cells are created by the controlled production of ROS via NOX-2, DUOX-1, and mitochondria and the expression of a variety of antioxidant systems, including superoxide dismutases, peroxiredoxins, and glutaredoxins. Indeed, the available data suggest that ROS play crucial roles in T-cell biology. First, ROS participate in activation-induced cell death and hence in the termination of the immune response. Several different sources of ROS appear to be involved in this process. It seems that DUOX-1 produces H_2_O_2_ that amplifies proximal TCR signaling, mitochondrial Complex I releases O_2_^•-^ that triggers the expression of FasL, and NOX-2 generates H_2_O_2_ that assists the execution of the apoptotic program. Second, ROS appear to be involved in the differentiation of T cells but dispensable for T-cell activation or proliferation. NOX-2-derived ROS direct the differentiation of T cells from the T_h_17 to the T_h_2 phenotype, at least in mice. Overall, under physiological conditions, there is an equilibrium between ROS and antioxidants, both in the tissue microenvironment and intracellular compartments, thus allowing normal T-cell responses. However, this delicate balance is disturbed in several diseases, such as systemic sclerosis, rheumatoid arthritis, AIDS, and cancer. The better understanding of the spatiotemporal dynamics and regulation of ROS production and elimination in T cells and their microenvironment is essential for the development of more effective treatments against various immune-mediated diseases.

Despite the remarkable progress in recent years, still much remains to be discovered about the function of ROS in T-cell biology. New experimental techniques should foster considerable advances in several areas of research. To address the compartmentalized nature of signaling ROS, methods for the highly sensitive spatial real-time measurement of ROS levels in living cells are desirable. The most promising approach in this field appears to be the combination of novel photostable, specific, reversible ROS probes [78] with the high-resolution microscopy of living cells [79]. The imaging flow cytometry [80], which combines the high throughput and populational analysis capabilities of traditional flow cytometry with the spatial resolution of microscopy, may also become particularly helpful. In order to discriminate between ROS produced by T cells and phagocytes, T-cell specific knockout mice for NOX-2, DUOX-1, and other ROS-producing enzymes are indispensable. Methods to efficiently and stably downregulate these proteins in primary human T cells without inducing oxidative stress, cell death, and cell preactivation are also essential. The microfluidic [81] delivery of shRNAs or CRISPRs [82] is amongst the most promising approaches. The same technique may be applied to knockdown peroxiredoxins, the major counterbalance to NOX enzymes. To understand the role of mitochondrial ROS, the organelle-targeted antioxidants [83] seem to be almost the exclusive tool, as it is not possible to inhibit the mitochondrial ROS-producing electron transport chain without affecting mitochondrial ATP production, calcium uptake, and metabolic functions. Finally, only few molecular targets of ROS in T cells are currently known [68]. The development of simple and reliable methods to measure protein sulfenylation [84], including the probes for real-time microscopy [85], would allow to investigate this new exciting area of research.

## Abbreviations

AICD: Activation-induced cell death
BHA: Butylated hydroxyanisole
CuZnSOD: Copper-zinc superoxide dismutase
DCs: Dendritic cells
DCFDA: Dichlorofluorescin diacetate
DHE: Dihydroethidium
DUOX: Dual oxidase
gp91^phox^: Phagocytic NADPH oxidase glycoprotein of 91 kDa
H_2_O_2_: Hydrogen peroxide
mito-roGFP: Mitochondrial reduction-oxidation sensitive green fluorescent protein
MnSOD: Manganese superoxide dismutase
NAC: N-acetyl cysteine
NOX: NADPH oxidase
O_2_^•-^: Superoxide
p47^phox^: Phagocytic NADPH oxidase protein of 47 kDa
PRX: Peroxiredoxin
RISP: Rieske iron-sulfur protein
ROS: Reactive oxygen species
TCR: T-cell receptor
TRX: Thioredoxin
